# CD34+ derived macrophage and dendritic cells display differential responses to paraquat

**DOI:** 10.1016/j.tiv.2021.105198

**Published:** 2021-09

**Authors:** Leonie F.H. Fransen, Martin O. Leonard

**Affiliations:** Toxicology Department, Centre for Radiation, Chemical and Environmental Hazards, Public Health England, Harwell Campus, OX11 0RQ, UK

**Keywords:** Myeloid, Lung, Transcriptomics, TempO-Seq

## Abstract

Paraquat (PQ) is a redox cycling herbicide known for its acute toxicity in humans. Airway parenchymal cells have been identified as primary sites for PQ accumulation, tissue inflammation and cellular injury. However, the role of immune cells in PQ induced tissue injury is largely unknown. To explore this further, primary cultures of human CD34+ stem cell derived macrophages (MC^cd34^) and dendritic cells (DC^cd34^) were established and characterised using RNA-Seq profiling. The impact of PQ on DC^cd34^ and MC^cd34^ cytotoxicity revealed increased effect within DC^cd34^ cultures. PQ toxicity mechanisms were examined using sub-cytotoxic concentrations and TempO-seq transcriptomic assays. Comparable increases for several stress response pathway (NFE2L2, NF-kB and HSF) dependent genes were observed across both cell types. Interestingly, PQ induced unfolded protein response (UPR), p53, Irf and DC maturation genes in DC^cd34^ but not in MC^cd34^. Further exploration of the immune modifying potential of PQ was performed using the common allergen house dust mite (HD). Co-treatment of PQ and HD resulted in enhanced inflammatory responses within MC^cd34^ but not DC^cd34^. These results demonstrate immune cell type differential responses to PQ, that may underlie aspects of acute toxicity and susceptibility to inflammatory disease.

## Highlights

• Paraquat induces inflammatory and oxidative events in immune cells.
• Paraquat prompts selective induction of several pathways in dendritic cells.
• Paraquat and dust mite co-exposure enhances inflammatory response in macrophages.
• These results provide insight into paraquat mechanisms of toxicity.

## List of abbreviations

**Table:** 

CTL	control
DC	dendritic cell
DC^cd34^	CD34+ derived dendritic cell
FC	fold change
F.O·C	fold over control
HD	house dust mite
IEG	immediate early genes
infMAC	inflammatory macrophage
infDC	inflammatory dendritic cell
IPA	ingenuity pathway analysis
MAPK	mitogen-activated protein kinase
MC	macrophage
MC^cd34^	CD34+ derived macrophage
LDH	lactate dehydrogenase
PBS	phosphate buffered saline
PQ	paraquat
RPKM	reads per kilobase million
SEM	standard error of the mean
TLR	toll-like receptor
UPR	unfolded protein response

## 1 Introduction

Paraquat (PQ) is a broad-spectrum herbicide known to be highly toxic to humans (Dinis-Oliveira et al., 2008; Subbiah and Tiwari, 2021). While it is currently banned within the European Union, in much of the rest of the world it is still commonly used. Upon ingestion, it results in systemic toxicity with acute mortality due to multiple organ failure and delayed mortality due to pulmonary fibrosis (Subbiah and Tiwari, 2021). Higher levels of PQ within the lung have been attributed to distal airway epithelial cell accumulation due to active absorption, suggested to be mediated through polyamine transporter expression (Dinis-Oliveira et al., 2008). While studies have demonstrated competitive inhibition of PQ uptake by polyamines (Hoet and Nemery, 2000; Silva et al., 2015), some *in vivo* work suggest additional mechanisms may be involved (Dunbar et al., 1988). The intracellular molecular mechanism of toxicity of PQ involves the reduction of PQ^2+^ ions as the initial step in the ability of this compound to act as a redox cycler, generating superoxide and subsequent H_2_O_2_. These oxidative chemical species cause cellular injury due to oxidation of lipids, proteins and DNA (Dinis-Oliveira et al., 2008). The reduction of PQ to generate superoxide within human cells requires enzymatic activity, and has been demonstrated for a number of reductase and oxidase enzymes, including mitochondrial complex I (Cochemé and Murphy, 2008), NADPH oxidases (NOX1–2; Cristóvão et al., 2009), nitric oxide synthase (NOS; Day et al., 1999), and cytochrome P450 oxidoreductase (POR; Han et al., 2006). Recently, a CRISPR-based knockout library screen identified POR as the primary mediator for PQ induced superoxide generation and toxicity in immortalized human T lymphocyte cells (Reczek et al., 2017).

Primary tissue responses to PQ exposure involve a prominent inflammatory response. Within the lung recruitment of innate immune cells such as polymorphonuclear leukocytes, NK-cells and macrophages occur (Dinis-Oliveira et al., 2008; Wu et al., 2020). While it is likely that inflammatory processes are initiated secondary to tissue injury from PQ exposure, there is also convincing evidence that PQ can have a direct effect on inflammatory cascades within resident structural and immune cells, possibly contributing to excessive responses. These include redox sensitive signalling pathways such as NF-κB and mitogen-activated protein kinase (MAPK) activation (Subbiah and Tiwari, 2021).

Macrophages are central regulators of tissue homeostasis and have been examined for their role in the response to PQ. Freshly isolated rat primary alveolar macrophages and type II pneumocytes exposed to PQ were highly sensitive to DNA strand breakage and base oxidation compared to other cell types (Dušinská et al., 1998; Petrovská and Dušinská, 1999). Alveolar macrophages were also observed to respond to systemic PQ exposure *in vivo* by increasing expression of the cystine/glutamate transporter SLC7A11, which is essential for maintaining intracellular glutathione and extracellular cystine/cysteine redox balance (Kobayashi et al., 2012). It has also been observed that PQ directly induced inflammatory mediators IL-8 and TNFα in human macrophage cell lines (Veríssimo et al., 2017). Furthermore it has also been suggested that inflammatory cells including macrophages play an active role in PQ induced pulmonary fibrosis development through the release of proteolytic enzymes (Schoenberger et al., 1984; Fukuda et al., 1985), or through the activation and recruitment of fibroblasts (Schoenberger et al., 1984; Conning et al., 1969). Importantly this has been suggested as independent of epithelial injury (Bus and Gibson, 1984). In addition, treatment of murine bone marrow derived macrophages with PQ resulted in an increased inflammatory cytokine IL-6, while airway epithelial cells did not respond (Hu et al., 2017). The authors suggest that such inflammatory signals induced by PQ contribute to the development of pulmonary fibrosis after chronic exposure.

Despite some evidence for the role of macrophages in the response to PQ induced tissue injury and inflammatory cascade, there has been no comprehensive evaluation of the response to PQ exposure in human primary cells. This is even more evident for other immune cells of myeloid origin such as dendritic and mast cells. While PQ has been observed to augment allergic mast cell degranulation and was suggested as a possible exacerbation risk for allergic conditions (Sato et al., 1998), there has been no comprehensive assessment of the effects of PQ on dendritic cells. As these cells are central to allergic development, insight in to the direct effect of PQ on these cells would provide clarity on whether PQ may pose a risk for allergic conditions, as has been suggested anecdotally (Cha et al., 2012; Díaz-Criollo et al., 2020; Hoppin et al., 2009). Therefore, in this study, we examined the global transcriptomic response of human primary CD34+ derived macrophage (MC^cd34^) and dendritic cells (DC^cd34^) in the absence and presence of the common allergen house dust mite (HD).

## 2 Methods

### 2.1 CD34+ stem cell expansion and myeloid differentiation

**Fig. 1:**
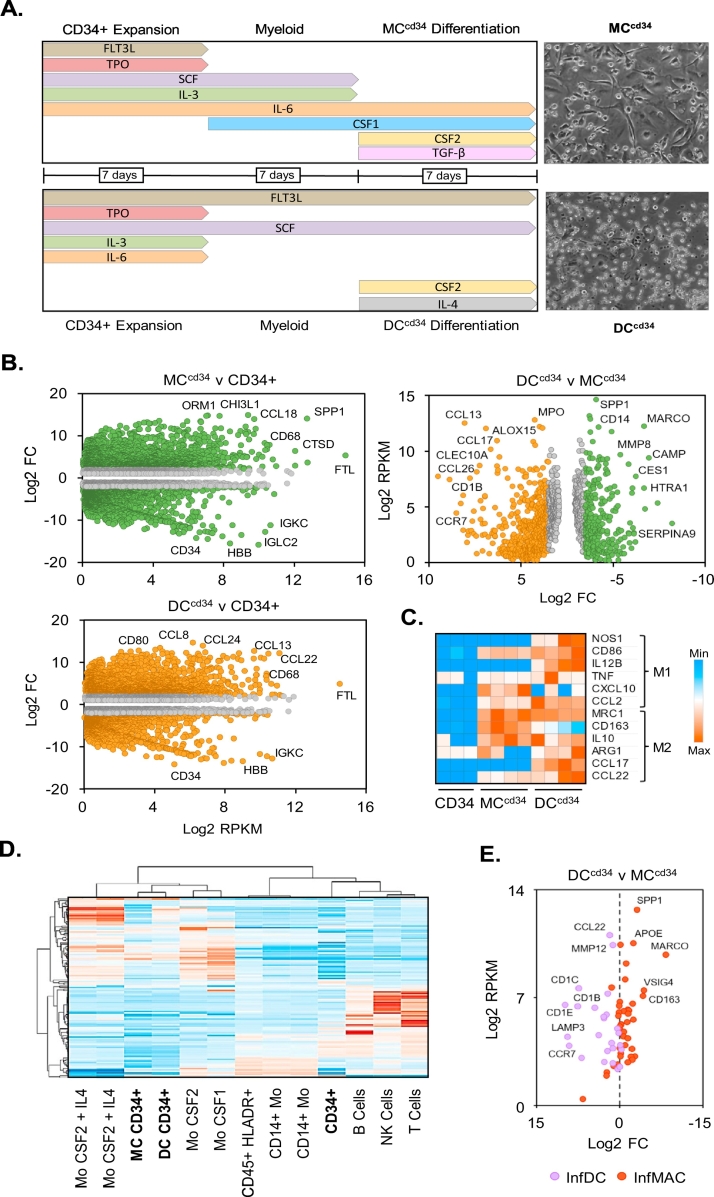
CD34+ macrophage and dendritic cell differentiation. Human bone marrow derived CD34+ cells were differentiated along a myeloid trajectory to macrophage and dendritic cell phenotypes as displayed, including morphological assessment (A). MC^cd34^ and DC^cd34^ cultures were examined for differences in gene expression compared to CD34+ stem cell undifferentiated cultures using RNA-Seq and displayed as volcano plots. Significantly regulated genes (>2-fold change with q value < 0.05) are highlighted as green (MC^cd34^) or orange (DC^cd34^). (B). M1 and M2 type macrophage markers were examined with cell populations and were presented as heatmap of log2 counts (C). Comparison of CD34+ cell type RNA-seq profiles (highlighted in bold) to published transcriptomic data was carried out using hierarchical clustering and presented as heatmap (D). Markers of inflammatory macrophage (InfMAC) and dendritic (InfDC) cells were examined within MC^cd34^ and DC^cd34^ RNA-seq data and displayed as log2 fold change v max RPKM values (E).

Bone marrow derived CD34+ stem cells were obtained from 5 different donors (Table S1; STEMCELL Technologies, Grenoble, France) and expanded for 1 week using Stemspan Media containing human serum albumin (0.05% w/v), penicillin/streptomycin (100 U/ml), human FLT3L (50 ng/ml), TPO (50 ng/ml), SCF (50 ng/ml), IL-6 (20 ng/ml) and IL-3 (20 ng/ml) on low attachment plates. Cells were further differentiated along the myeloid lineage according to the protocol outlined in Fig. 1A for a further week using CSF1 (25 ng/ml) for the macrophage lineage. For the final week of differentiation in 24 well plates, cells were incubated in media further supplemented with CSF1 (50 ng/ml), CSF2 (50 ng/ml) and TGF-b (2 ng/ml) for macrophage, and CSF2 (20 ng/ml) and IL-4 (20 ng/ml) for dendritic lineages.

### 2.2 Cell treatment and viability assessment

MC^cd34^ and DC^cd34^ cultures seeded at an initial density of 5 × 10^4^ cells/ml in 48 well plates were treated with paraquat (Merck) or busulfan (Merck) for 24 h. Select treatments were also carried out with house dust mite soluble extracts (Stallergenes Greer, US). Cellular resazurin reduction and lactate dehydrogenase (LDH) into the media were used as measures of viability. Cell culture media LDH content was indicative of necrotic cellular leakage and determined after centrifugation using a commercial kit (Merck; Cat# 4744926001) according to manufacturer's instructions. Resazurin reduction as a measure cellular and mitochondrial reductase activity was carried out as previously described (Meldrum et al., 2016). Briefly, cells were washed in PBS and incubated 44 μM resazurin in cell culture media (Cat# R7017, Merck) for 1 h at 37C. Fluorescence was then detected at 540 nm excitation and 590 nm emission. Statistical significance compared to control values was carried out using one-way ANOVA and Fisher's LSD test in GraphPad Prism software Version 8.3.0 and results are expressed as mean ± standard error of the mean (SEM) unless otherwise stated.

### 2.3 RNA extraction and transcriptomic analysis

For RNA-seq analysis, after cell treatment total RNA was isolated using an RNeasy minikit (Qiagen, Valencia, CA). RNA quantity was determined using the nanodrop platform (Thermo Scientific) and quality determined using an Agilent 2100 Bioanalyzer. Samples with an RIN above 8.0 were used for TruSeq™ library preparation (Illumina, San Diego, USA). RNA processing and sequencing was carried out using 150PE sequencing method on the Illumina HiSeq™ 2000 platform (BGI, Hong Kong). Raw sequence data was processed to remove adaptor sequences, contamination and low-quality reads. Up to 40 million clean reads per sample were annotated to human GRCh38 reference genome and counts processed for relative gene expression using CLC Genomics browser software (CLCBIO, Aarhus, Denmark). Statistically significant gene expression across CD34+ and differentiated lineages was considered using Benjamin Hochberg adjusted *p* values < 0.05. For TempO-Seq analysis of mRNA expression levels for 3565 gene probe sets for genes involved in toxicological responses were assessed as previously described (Limonciel et al., 2018). After treatment cells were lysed using 2× lysis buffer and samples outsourced and processed for quantification at Bioclavis (Glasgow, UK). Raw counts for each probe-set and sample were then analysed for differential regulation with cell treatment using DESeq2 version 1.30.0 software within an R environment (Love et al., 2014). Normalised counts were obtained using the EstimatedSizeFactors function within DESeq2. Differential expression was calculated using the Wald test statistical significance was called using Benjamin Hochberg adjusted *p* values < 0.05.

## 3 Results

### 3.1 Characterisation of CD34 derived macrophage and dendritic cell populations

Human primary bone marrow derived CD34+ stem cells from 4 different donors were differentiated towards macrophage and dendritic cell lineages according to Fig. 1A. CD34+ derived macrophage (MC^cd34^) differentiation was driven by IL-6, CSF1, CSF2 and TGF-β, while CD34+ derived dendritic (DC^cd34^) differentiation was driven by FLT3L, SCF, CSF2 and IL-4. Morphological differences were observed between the two lineages, with MC^cd34^ displaying a more adherent and elongated profile compared to a more non-adherent state in DC^cd34^ cultures (Fig. 1A). RNA-seq was used to characterise the mRNA profiles of these two differentiated cell types (Fig. 1B). Comparison of differentiated MC^cd34^ and DC^cd34^ cultures to CD34+ stem cell origin cultures revealed similar increased levels of mononuclear phagocyte markers such as FTL and CD68 (Chistiakov et al., 2017), indicating a common myeloid differentiation trajectory. Conversely, there was a marked reduction in CD34 confirming a trajectory away from a stem cell origin. Interestingly there was also a reduction in plasma immunoglobulin and globin genes on myeloid differentiation, indicating the presence of mixed stem cell or pluripotency potential towards non-myeloid lineages within the CD34+ stem cell population. Among the most highly differentially regulated genes on MC^cd34^ v CD34+ cell analysis was CHI3L1 and SPP1, typically associated with macrophage function. Similarly, upon DC^cd34^ v CD34+ cell analysis the antigen presentation associated gene CD80 was among the most highly regulated. A comparison was also carried out to highlight the differences between MC^cd34^ and DC^cd34^ cultures (Fig. 1B; right panel). This demonstrated macrophage (*e.g.* MARCO, CAMP) and dendritic cell (*e.g.* CCR7, CLEC10A) marker specific expression within MC^cd34^ and DC^cd34^ cultures respectively. MC^cd34^ and DC^cd34^ differentiation was confirmed using a separate set of donors and different transcriptomic method of characterisation, demonstrated highly significant correlation (Fig. S6B). To characterise these cell lineages in further detail, we examined prototypical inflammatory disease associated M1 and M2 macrophage profile markers within CD34+ derived populations. Inflammatory MCs can become activated by exposures to for instance mediators released by injured tissues, directing the development of inflammatory (M1) and anti-inflammatory (M2) MC populations (Tsou et al., 2007; Laskin et al., 2019). Excessive M2 activation after acute injury may result in chronic disease such as fibrosis and asthma (Laskin et al., 2019). While, these M1 and M2 markers demonstrated differential expression compared to CD34+ cells, their profile of expression did not corelate to either MC^cd34^ or DC^cd34^ cultures. To further define the cell type profile of these cultures, alignment to human immune cell transcriptomic data from tissue and *in vitro* derived origin was carried out (Fig. 1D). Cluster analysis of highly dispersed and cell type marker expression (as described in methods) revealed the highest similarity of MC^cd34^ or DC^cd34^ cultures to monocytes differentiated to macrophage like cells using CSF2 and CSF1. MC^cd34^ and DC^cd34^ did not cluster closely to lymphocyte, CD14+ monocytes or lung derived myeloid cell populations (lin- CD45+ HLADR+). Inflammatory macrophage and dendritic cells derive from monocytes and are active participants in ongoing immune processes within diseased tissues (Segura et al., 2013). They are distinct from resident macrophage and dendritic cells within mucosal surfaces and display unique profiles of gene expression. When mRNA markers used to define these cells (Segura et al., 2013), were examined within our MC^cd34^ and DC^cd34^ datasets, separation to inflammatory macrophage and dendritic cell types was readily apparent (Fig. 1E).

### 3.2 Paraquat exposure results in lineage dependent cellular responses

**Fig. 2:**
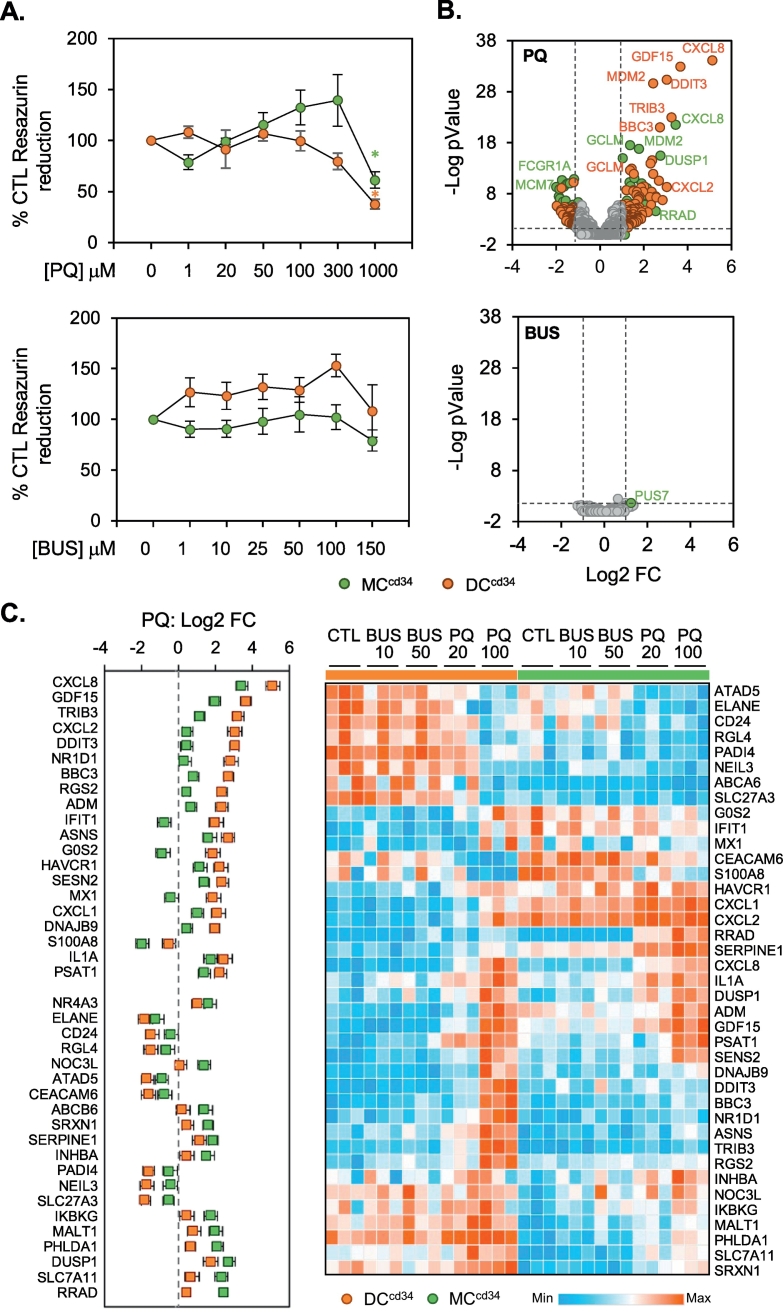
Toxicological assessment in response to chemical exposure. MC^cd34^ and DC^cd34^ cultures were exposed to varying doses of paraquat (PQ) or busulfan (BUS) for 24 h and toxicological assessment was carried using resazurin reduction (A). Results were expressed as mean percentage change (%) ± standard error of the mean (SEM) (A). Statistical differences compared to control (*p* < 0.05 shown as *, ANOVA). Analysis of mRNA transcript changes after 24 h was also assessed for PQ (100 μM) and BUS (50 μM) in each cell type using tempo-seq and displayed as a volcano plot of fold change v pValue (B). Significant (FC > 2/−2 + Adj pVal<0.05) DC^cd34^ responses are highlighted in orange, while MC^cd34^ are displayed in green (B). Those PQ altered genes within the TempO-Seq data, which showed the largest differences in fold change responses between cell types are displayed as a boxplot (C), with corresponding normalised mRNA molecule counts per sample for these same genes across all cell treatments are displayed as a heatmap (C).

**Fig. 3:**
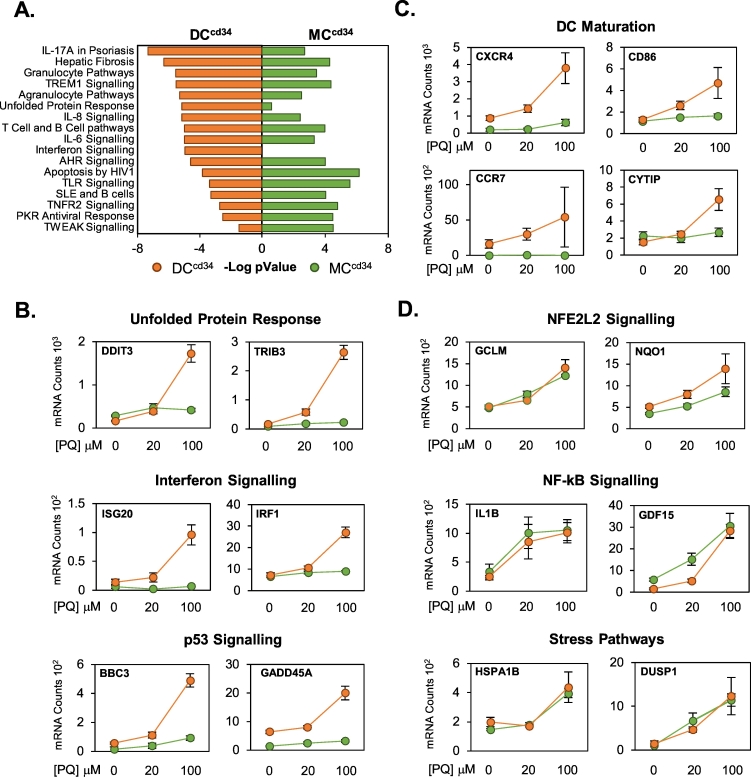
Paraquat causes cell type dependent transcriptomic responses. MCcd34 and DCcd34 cultures were exposed to paraquat (PQ; 100 μM) for 24 h and transcriptomic changes were analysed using TempO-Seq. Gene expression data identified as statistically significantly altered in each cell type compared to control were processed using IPA software for significantly regulated pathways (A). Normalised mRNA molecule counts from TempO-Seq data was displayed for select groupings of genes (mean ± standard error of the mean (SEM)) to allow comparison of pathway responses between cell types (B–D). Dendritic (DC) maturation markers were also included (B).

The cytotoxic responses of MC^cd34^ and DC^cd34^ to PQ exposure was assessed using resazurin reduction and LDH release assays (Fig. 2A, Fig. S1). In contrast to PQ, which is a redox cycler, busulfan is an alkylating agent and was included as a comparator compound with a different molecular mechanism of cellular injury. PQ induced a dose dependent alteration in resazurin reduction and LDH release, which was more pronounced in DC^cd34^ than MC^cd34^ indicating an increased sensitivity within the dendritic lineage (Fig. 1A). Busulfan resulted in a general increase in resazurin reduction in DC^cd34^ cells, which was not observed with PQ (Fig. 1A). LDH release displayed a difference between both cell types. Solubility issues prevented examination of busulfan at higher doses. Further examination of PQ cellular responses was carried out at doses (20–100 μM) judged to be below gross toxicological effects in order to capture stress response pathway changes in the absence of active cytotoxic processes. These concentrations are similar to those found within the plasma of patients admitted to hospital after PQ ingestion (26.67 μg/ml; 104 μM; Hong et al., 2014). Transcriptomic changes in response to chemical exposure for 24 h was assessed using TempO-Seq technology (Fig. 2B,C). PQ at 100 μM resulted in differential expression of 227 genes in DC^cd34^ and 223 genes within MC^cd34^ cultures, while busulfan at 50 μM resulted in only 1 dysregulated gene within MC^cd34^ cells (Fig. 2B). Within these selected gene sets, differences between DC^cd34^ and MC^cd34^ responses to PQ were presented as priority lists of those genes with preferential fold change induction for each cell type (Fig. 2C; left panel). CXCL8, GDF15 and TRIB3 were among the genes preferentially induced in DC^cd34^ while RRAD, SLC7A11 and DUSP1 were preferentially induced in MC^cd34^. These set of genes were investigated further, where gene count levels were displayed within a heatmap for both busulfan and PQ treatments (Fig. 2C; left panel). This analysis allows us to see that some genes whose expression appeared to be preferentially induced within a single cell type based on fold change, displayed similar absolute count levels of gene expression after PQ treatment, including GDF15 and CXCL8. This apparent preferential fold change-based effect can be attributed to different control levels of genes between cell types and is further exemplified for genes such as CXCL1 and CXCL2. There are however clear instances of expression profiles of genes where CTL count levels between cell types are similar and there remains preferential induction in a particular cell type, for example PQ induced DDIT3 and TRIB3 in the DC^cd34^ population (Fig. 2C). Pathway analysis was also carried out on those differentially regulated genes by PQ in both cell types to identify processes common to but also preferentially activated by chemical exposure (Fig. 3A). Inflammatory pathways were in general the most significantly identified and were similarly regulated between both cell types. However, DC^cd34^ cultures responded preferentially for unfolded protein response and interferon signalling pathways when compared to MC^cd34^ (Fig. 3A). This preferential pathway activation in DC^cd34^ is confirmed when selected pathway genes are examined (Fig. 3B) and reveal dose dependent effects in response to PQ. In addition, p53 pathway genes were preferentially activated in DC^cd34^ cultures. Importantly, dendritic cell maturation markers were also selectively induced in DC^cd34^ compared to MC^cd34^ (Fig. 3C), suggesting the potential that PQ may direct antigen presentation and adaptive immunity responses in exposure settings and disease states. Interestingly, not all stress response pathways responded with differential effects between cell types. Similar responses for NFE2L2, NF-kB, HSF and immediate early genes (IEG) were observed between DC^cd34^ and MC^cd34^ exposures (Fig. 3D), indicating that preferential responses in DC^cd34^ may be attributable to cellular differentiation dependent response capability, rather than differences in perceived chemical challenge.

### 3.3 Combined paraquat and house dust mite allergen effects on CD34+ derived cells

**Fig. 4:**
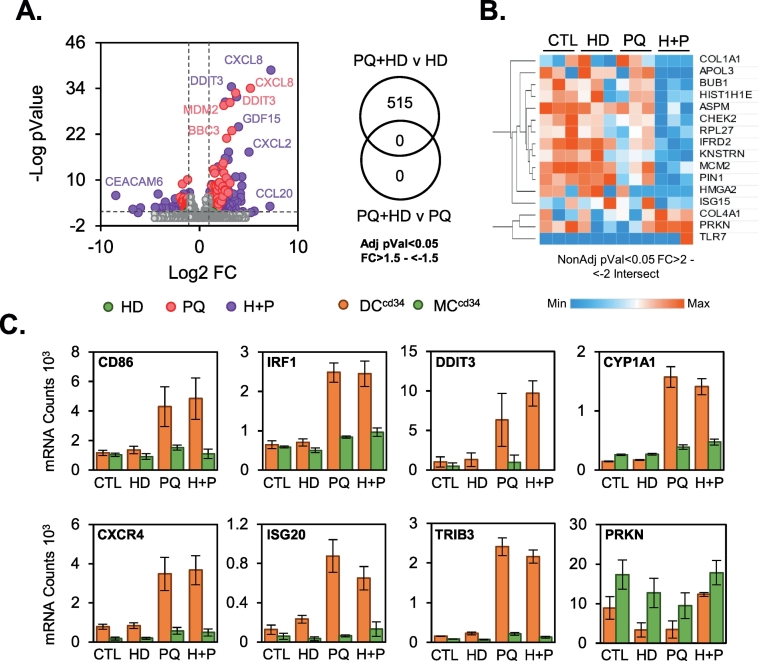
Effect of house dust mite co-exposure on paraquat responses in DC^cd34^. DC^cd34^ cultures were exposed to paraquat (PQ; 100 μM) alone or in combination with house dust mite (HD; 25 μg/ml) for 24 h and transcriptomic changes were analysed using TempO-Seq. Differentially regulated genes (FC > 2/−2 + Adj pVal<0.05) over control were displayed as a volcano plot, and numbers of genes within select comparisons indicated within the Venn diagram (A). Analysis using less stringent statistics (NonAdj pVal) was also applied to the TempO-Seq data. Those genes displaying enhanced PQ + HD changes above both treatments alone were displayed as a heatmap of normalised mRNA molecule count values (B). Select genes representing DC^cd34^ selective responses to PQ were displayed for the effect of co-treatment with HD as mRNA counts (mean + SEM) as well as a comparison to effects in DC^cd34^ treatments (C).

The ability of PQ to modulate inflammatory signalling within DC^cd34^ and MC^cd34^ populations and the potential sensitisation effects within DC^cd34^ led us to examine whether there were any modifying effects upon co-exposure with the common allergen house dust mite (HD; Meldrum et al., 2018). HD treatment (25 μg/ml) either alone or in combination with PQ (100 μM) did not result in significant alterations in cytotoxicity (Fig. S5 – LDH) for either cell type. Examination of TempO-Seq analysis of mRNA levels from DC^cd34^ exposures after 24 h revealed a similar level of expression between PQ alone and PQ + HD (Fig. 4A; left panel). HD alone did not induce any significant changes in gene expression in DC^cd34^. To identify any additive or more than additive effects resulting from co-exposures, we selected those genes commonly differentially regulated when PQ + HD v PQ was compared to PQ + HD v HD treatments. No genes fell into this category (Fig. 4A; right panel). Further analysis to identify combined exposure effects was carried out using a more relaxed statistical application, where unadjusted *p* value criteria was used instead of adjusted p value. This resulted in 16 commonly regulated genes (Fig. 4B) with the majority displaying a reduction in count levels compared to control and single treatments. The relevance of these changes is uncertain given the lack of statistically significant results. There were no apparent differences in DC^cd34^ selective PQ induced pathways, including DC maturation (CD86, CXCR4), interferon (IRF1, ISG20) and UPR (DDIT3, TRIB3) when PQ + HD was compared to PQ alone (Fig. 4C). These results indicate combined exposure with HD has little impact on PQ induced effects in DC^cd34^ cultures.

**Fig. 5:**
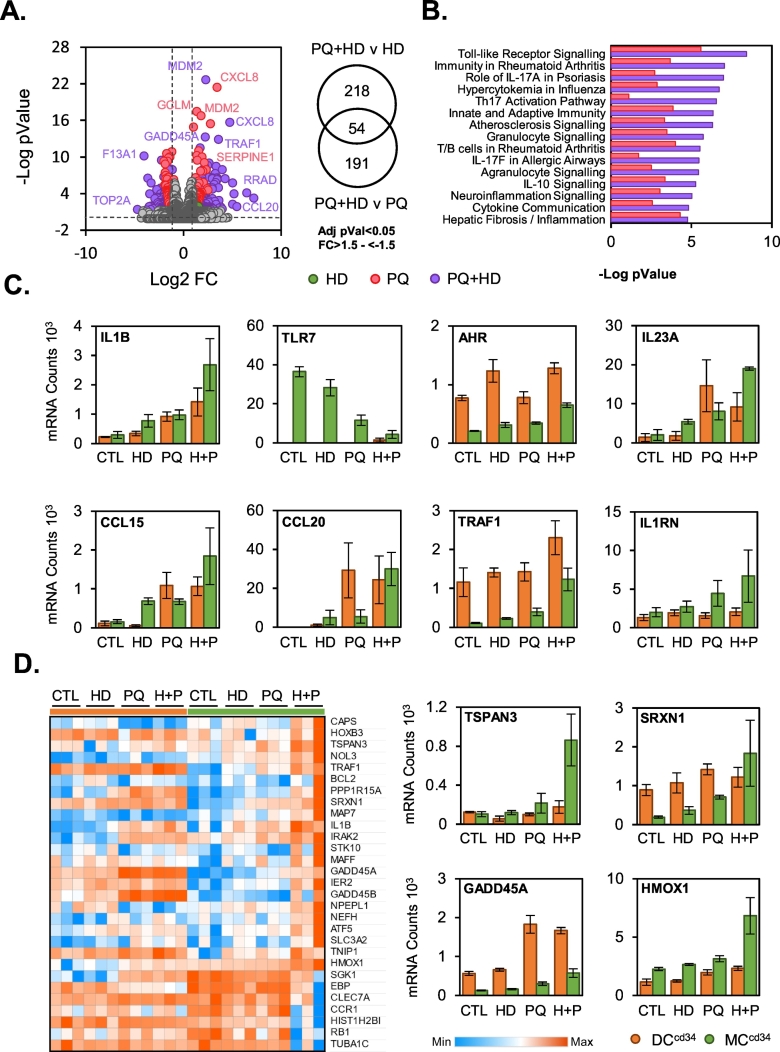
House dust mite co-exposure alters paraquat responses in MC^cd34^. MC^cd34^ cultures were exposed to paraquat (PQ; 100uM) alone or in combination with house dust mite (HD; 25μg/ml) for 24 h and transcriptomic changes were analysed using TempO-Seq. Differentially regulated genes (FC > 2/−2 + Adj pVal<0.05) over control were displayed as a volcano plot, and numbers of genes within select comparisons indicated within the Venn diagram (A). Gene expression data identified as statistically significantly altered with each treatment compared to control were analysed using IPA software for significantly regulated pathways (B). Results are displayed as -Log10 pValue. Those genes identified as enhanced with combined PQ + HD treatment (Venn diagram intersect; 54 genes) in MC^cd34^ cells were further prioritised and select genes were displayed, including the effects within DC^cd34^ cultures (C). A heatmap of normalised mRNA count values was also included to display the top 30 enhanced genes within this comparison (D).

Examination of TempO-Seq analysis of mRNA levels from MC^cd34^ exposures after 24 h revealed that HD had minimal impact on gene expression, differentially regulating 2 genes. It did however result in an increase number of regulated genes (440 genes) when given in combination with PQ (HD + PQ) when compared to PQ alone (223 genes; Fig. 5A). Pathway analysis TempO-Seq data for PQ + HD and PQ responses compared to control revealed a clear increase in significance for a broad range of inflammatory signalling categories in PQ + HD when compared to PQ alone effects (Fig. 5B). When we examined those genes commonly regulated across PQ + HD v PQ and PQ + HD v HD comparisons, as additive or more than additive responses to co-treatment, we found 54 genes (Fig. 5A; right panel). A large proportion of these genes are involved in inflammatory processes, examples of which are displayed in Fig. 5C,D. When mRNA count levels for these genes were compared to responses to the same treatments in DC^cd34^, maximal levels for PQ + HD in MC^cd34^ did not go beyond CTL or PQ induced levels in DC^cd34^. This may indicate that a lack of combinatorial PQ + HD effects in DC^cd34^ could be due to a maximal activation of select signalling events in this cell type compared to MC^cd34^. However, there were some clear MC^cd34^ specific PQ + HD combinatorial effects such as IL1B, TSPAN3 and HMOX1, that went beyond levels observed within DC^cd34^ cultures (Fig. 5C), indicating a unique macrophage lineage responsiveness.

## 4 Discussion

Myeloid cells such as macrophages (MCs) and dendritic cells (DCs) play an important role in innate defence mechanisms in most human tissues including the lung (Janeway et al., 2001; Kelly and O'Neill, 2015; Kopf et al., 2015). The ability to differentiate CD34+ hemopoietic stem cells *in vitro* towards DC^cd34^ and MC^cd34^ (Rosenzwajg et al., 1996; Caux et al., 1996; Young et al., 1995; Clanchy and Hamilton, 2013) recapitulates *in vivo* differentiation trajectories typically observed in inflammatory disease states. In this study we characterised MC^cd34^ and DC^cd34^ lineages to have similar transcriptomic expression to inflammatory macrophage and dendritic cells respectively (Segura et al., 2013). Expression of genes including MARCO and CAMP in MC^cd34^ cells and CCR7 and CD1B in DC^cd34^ indicate functional macrophage and dendritic cell differentiation respectively (Janeway et al., 2001; Kelly and O'Neill, 2015; Kopf et al., 2015; Rosenzwajg et al., 1996; Caux et al., 1996; Young et al., 1995; Clanchy and Hamilton, 2013).

Having established the ontogeny of our test cultures, we next set out to understand how PQ exposure may alter their function and the molecular mechanisms involved. We demonstrate that PQ did not significantly reduce viability in DC^cd34^ and MC^cd34^ cultures at concentrations below 1 mM. The decrease in resazurin reductase activity and increase in supernatant LDH observed at 1 mM was more pronounced in DC^cd34^ cultures than MC^cd34^. We also tested the alkylating agent busulfan and did not see any significant changes in viability at the concentrations tested. To understand how PQ may differentially impact these two cell types, we next examined global transcriptomic responses. PQ induced near identical changes in NFE2L2, NF-kB, HSF and IEG stress response pathway dependent gene expression (Fig. 3D). This would indicate consistent oxidative stress signalling responses across both cell types. However, there was a striking difference in the levels of gene expression for the stress response pathways p53 and UPR, which were activated in DC^cd34^ cultures but not in MC^cd34^. These differential effects indicate a selective responsiveness of DC^cd34^ to PQ that may underlie differences in viability observed in our study and may also underpin the selective activation of DC maturation markers observed in DC^cd34^.

There are a number of explanations for the differential effects observed between both cell types. The first is an increased inherent sensitivity to the same level of PQ exposure, reflective of differences in subcellular structures and pathway activation. We can explore this possibility by examining gene expression for components of each pathway selectively activated in DC^cd34^ cultures, within our RNA-Seq datasets (Fig. S2). We acknowledge this comparison does not determine the contribution of post-translational modifications or epigenetic changes that may underlie enhanced sensitivity. There was an absence of or only minor changes to the expression of UPR regulator genes PERK (EIF2AK3), IRE1 (ERN1), ATF6 and XBP1. There was also little difference in p53 pathway genes TP53 and MDM2. Both the IRF1 and NF-kB transcription factors (TF) have been observed to regulate IRF dependent genes identified within DC^cd34^ cultures (Forero et al., 2019; Espert et al., 2004). No differences in the levels of these TF components STAT1, IRF1, RELA, RELB or NFKBIA were observed in this study (Fig. S2).

Another explanation for the difference between cell types could centre on different levels of intracellular exposure. *In vitro* studies have suggested that PQ enters cells through polyamine transporters (Dinis-Oliveira et al., 2008; Hoet and Nemery, 2000). Gene expression levels of the primary polyamine transporters SLC3A2, SLC7A1, ATP13A3 or SLC47A1 were not significantly altered between cell types (Fig. S2). The transporter SLC7A2 and the SLC22A family of polyamine transporters displayed very low levels of expression. The SLC18B1 polyamine transporter displayed lower levels of in DC^cd34^ compared to MC^cd34^. This transporter has been observed within activated macrophages and is suggested to mediate vesicular accumulation of polyamines (Moriyama et al., 2020; Park et al., 2019). However, using mRNA levels as an indicator for the level of transporters has limitations as they might not accurately reflect the presence of transporter proteins, hampering comparison without further characterisation, so possible differences in intracellular PQ levels, including localised PQ accumulation are unknown at this stage. However, if we can assume that redox cycling activity of paraquat in our data are the primary trigger for NFE2L2 pathway activation, the observation that we have similar levels of PQ induced NFE2L2 gene expression in both cell types, would suggest that similar levels of cytosolic PQ are observed between cell types. Therefore, differences we observe between DC^cd34^ and DC^cd34^ cells may be due to other structural or functional variations between the cells, potentially involving more localised intracellular effects and/or organelle specific reactivity.

Recent insight into PQ toxicity mechanisms were revealed using a CRISPR library knockdown approach (Reczek et al., 2017). This screen identified POR, SLC45A4 (sucrose transporter) and ATP7A (copper transporter) as contributors to PQ induced cell death in Jurkat cells. POR and SLC45A4 expression were unaltered between DC^cd34^ and MC^cd34^ cultures. ATP7A is a copper efflux regulator and was suggested to contribute to cellular toxicity through reduced Cu dependent SOD1 activity and subsequent reduction of superoxide levels. Superoxide dismutase activity has been previously demonstrated to play an important role in reducing PQ induced toxicity (Filograna et al., 2016). This mechanism was further supported by a negative selection CRISPR screen, which identified knockdown of the copper transporter SLC31A1 (CTR1) and SOD1 sensitised cells to PQ injury (Reczek et al., 2017). Interestingly, SLC31A1 and SLC31A2 (CTR2) were downregulated in DC^cd34^ compared to MC^cd34^ cultures in our study, while the efflux transporters ATP7A and ATP7B were marginally upregulated (Fig. S3). No changes in the Cu transporter accessory proteins ATOX1 and COX17 were observed. Importantly, there was a large reduction in Cu dependent SOD2 gene levels in DC^cd34^ compared to MC^cd34^ cultures (Fig. S3), when combined with reduced Cu transport levels within DC^cd34^ would indicate a reduced capability to detoxify superoxide. There were no changes in SOD1 expression between cell types, and as SOD2 is located within the mitochondria, the possibility arises that increased PQ mediated superoxide levels are observed within the mitochondria of DC^cd34^ and contribute to selective activation of a group of signalling events including the UPR, p53 and IRF pathways.

The idea that PQ may be acting within the mitochondria is consistent with the ability of complex I to mediate PQ redox cycling and superoxide production (Karnati et al., 2013). We examined control gene expression differences for complex I components between both cell types and found no significant differences (Fig. S3). In addition to complex I, other targets for PQ induced superoxide have been identified (Dinis-Oliveira et al., 2008). No significant increases in these genes were observed within the DCs (Fig. S3). On the contrary, there was a reduction in CYBB (NOX2) and CYBA. There was also a small reduction in POR in DC^cd34^ *versus* MC^cd34^. Furthermore, there were little or no changes in the levels of NOX enzyme co-factors NCF1, NCF2 and NCF4 or other PQ targets NOX1 or NOS1. Interestingly though, there was an increase in another class of professional superoxide generating enzyme complex genes in DC^cd34^. These are the dual oxidase genes and are best characterised for their role in regulating anti-microbial responses within immune cells (Knaus, 2021). DUOX1 and DUOAX1 genes were absent from MC^cd34^ but were substantial expressed in DC^cd34^ (Fig. S3). DUOX2 was also absent form MC^cd34^ and present in DC^cd34^ but to a lesser extent. Proteins encoded by these genes are located at the plasma membrane and therefore may not be responsible for intracellular oxidative stress activation (Morand et al., 2009; Meitzler et al., 2013; van der Vliet et al., 2018).

Mitochondrial stress responses include changes in oxidative phosphorylation, regulation of inflammatory signalling, apoptosis and induction of the mitochondrial unfolded protein response (Patergnani et al., 2020; Münch, 2018). One of the most striking differences in PQ responsiveness between cell types was the DC^cd34^ selective induction of unfolded protein response genes including DDIT3 and TRIB3. It is therefore possible that UPR activation in DC^cd34^ cultures may be attributable to increased mitochondrial superoxide mediated protein misfolding as a consequence of reduced mitochondrial SOD activity. To distinguish such activation from endoplasmic reticulum detection of proteotoxic stress requires further study. Such a mechanism for inflammatory IRF mediated signalling through mitochondrial mechanisms is also a possibility (West et al., 2015).

As an increased production of ROS catalysed by *e.g.* NOX2 is a defence mechanism against pathogens for MCs, there are self-protective mechanisms against the potential oxidative stress (Wang et al., 2019; Virág et al., 2019). Activation of the NRF2 pathway was shown to be the main pathway to alleviate oxidative stress in inflammatory macrophages, upregulating anti-oxidant genes such as HMOX1 and NQO1 as well as GCLC and GCLM which are involved in glutathione synthesis (Wang et al., 2019; Tonelli et al., 2018). However, we did not see a difference in upregulation of the NRF2 pathway activation or antioxidant genes such as NQO1 and GCLM between MC^cd34^ and DC^cd34^, indicating protective mechanisms might not be causing the observed differences in response to PQ. In DCs, however, mitochondrial ROS production was shown to be required to induce CD8+ T cell responses (Oberkampf et al., 2018; Matsue et al., 2003), indicating that specific PQ induced increases in DC maturation (CXCR4, CCR7, CD86 and CYTIP) markers observed in DC^cd34^ might be related to innate biological functions within DC.

While PQ treatment alone showed selective activation of pathways such as UPR in DC^cd34^, these responses were not altered by HD in DC^cd34^ cultures. However, PQ responses were altered by HD in MC^cd34^, showing predominantly differential expression of inflammatory response genes. A proportion of those genes that displayed additive or more than additive activity within MC^cd34^ cultures displayed expression levels below control or PQ alone induced effects in DC^cd34^ cultures. This would indicate that part of the reason for a lack of modifying effect of HD on PQ in DC^cd34^ may be due to maximal expression (*e.g.* AHR). On the other hand, there is a clear enhancement in MC cultures that do not fall into this category (*e.g.* IL1B, TSPAN3, HMOX1) and would indicate an inherent sensitivity of MC^cd34^ to HD in the presence of paraquat. When we examine the levels of genes of known cellular receptor components of HD (Liu et al., 2005; Lundell et al., 2005), we identify the TLR4 co-factor CD14 as highly expressed in MC^cd34^ cultures compared to DC^cd34^ (Fig. S4). This was also the case for CLEC6A and CLEC4D, known receptors for other components of HD mixtures. This would suggest that enhanced responses in MC^cd34^ cultures may be due to differences in HD component inflammatory receptor profiles between cells. It is also interesting to note that these differences were not observed with HD alone, indicating that additional cellular stress signals are needed to manifest this inflammatory response.

In summary, we demonstrate that PQ has selective effects in DC^cd34^ cultures, including DC maturation marker expression, when compared to MC^cd34^. We also demonstrate that MC^cd34^ cultures display enhanced inflammatory responsiveness to HD and PQ co-treatments. The consequence for these differential sensitivities in cell types and the unique PQ response within DC^cd34^ cultures are novel and may have implications for our understanding of not only acute PQ toxicity but also inflammatory disease conditions such as allergy and asthma.

## Ethics approval and consent to participate

N/A.

## Consent for publication

Not applicable.

## Availability of data and materials

The datasets used and/or analysed during the current study are available from the corresponding author on reasonable request. RNA-Seq and TempO-Seq data are available on the Gene Expression Omnibus (GEO) database (Accession number GSE168794).

## Funding

The work was funded by the Marie Sklodowska-Curie Action-Innovative Training Network project in3, under grant no. 721975. This study is part funded by the National Institute for Health Research (NIHR) Health Protection Research Unit in Environmental Exposures and Health, a partnership between Public Health England and Imperial College London. The views expressed are those of the author(s) and not necessarily those of the NIHR, Public Health England or the Department of Health and Social Care.

## Authors' contributions

LFHF and MOL designed the experimental approach, carried out the work and analysed the resulting data. MOL acquired the funding and provided overall supervision. MOL and LFHF wrote the manuscript.

## Declaration of Competing Interest

The authors declare that they have no competing interests. The authors alone are responsible for the content and writing of the manuscript.

## Appendix A Supplementary data

Supplementary figures 1-6 Image 1

Supplementary table 1 Image 2
