# Supplementary material for: CD34+ derived macrophage and dendritic cells display differential responses to paraquat
Source: Toxicol In Vitro. 2021 Sep;75:105198. doi: 10.1016/j.tiv.2021.105198 (PMC8444090; doi:10.1016/j.tiv.2021.105198)
Supplement: Supplementary file 1 — Supplementary figures 1-6 [file mmc1.pptx]

## Slide 1
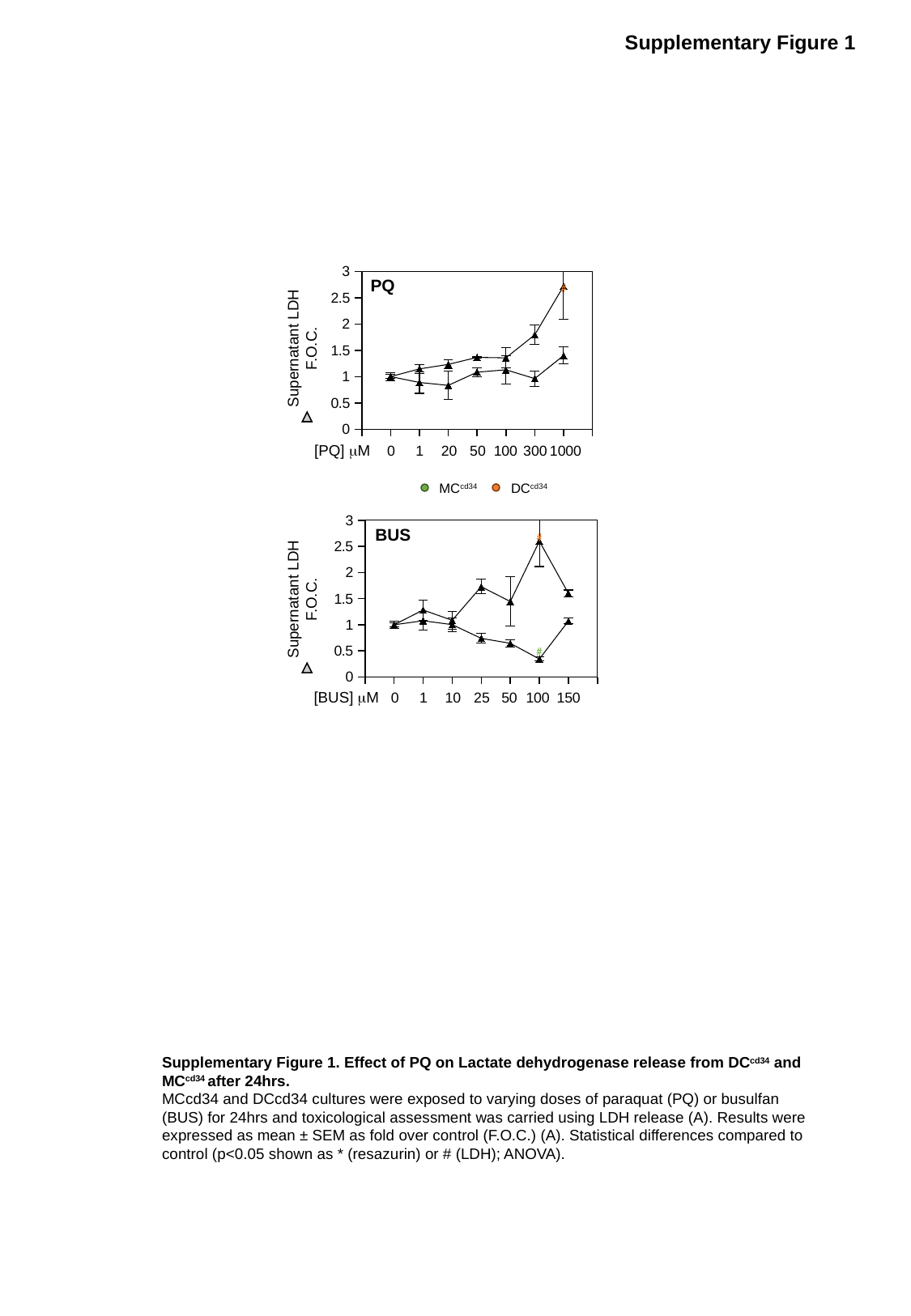

Supplementary Figure 1
### Chart
| Category | MC | DC |
|---|---|---|PQ
#
Supernatant LDH F.O.C.
[PQ] mM
0
1
20
50
100
300
1000
MCcd34
DCcd34
### Chart
| Category | MC | DC |
|---|---|---|BUS
#
Supernatant LDH F.O.C.
#
[BUS] mM
0
1
10
25
50
100
150
Supplementary Figure 1. Effect of PQ on Lactate dehydrogenase release from DCcd34 and MCcd34 after 24hrs.
MCcd34 and DCcd34 cultures were exposed to varying doses of paraquat (PQ) or busulfan (BUS) for 24hrs and toxicological assessment was carried using LDH release (A). Results were expressed as mean ± SEM as fold over control (F.O.C.) (A). Statistical differences compared to control (p<0.05 shown as * (resazurin) or # (LDH); ANOVA).

## Slide 2
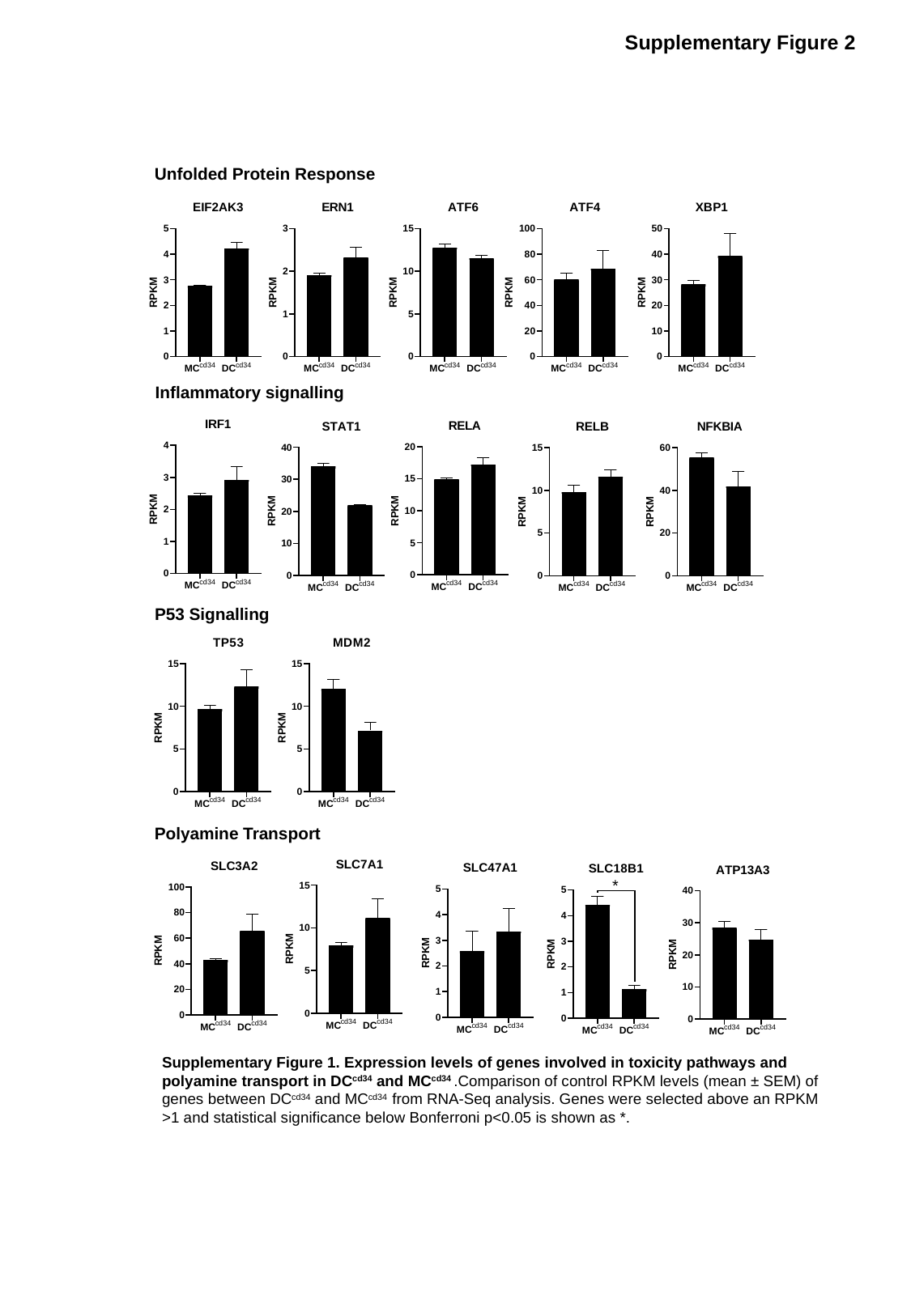

Supplementary Figure 2
Unfolded Protein Response
Inflammatory signalling
P53 Signalling
Polyamine Transport
Supplementary Figure 1. Expression levels of genes involved in toxicity pathways and polyamine transport in DCcd34 and MCcd34 .Comparison of control RPKM levels (mean ± SEM) of genes between DCcd34 and MCcd34 from RNA-Seq analysis. Genes were selected above an RPKM >1 and statistical significance below Bonferroni p<0.05 is shown as *.

## Slide 3
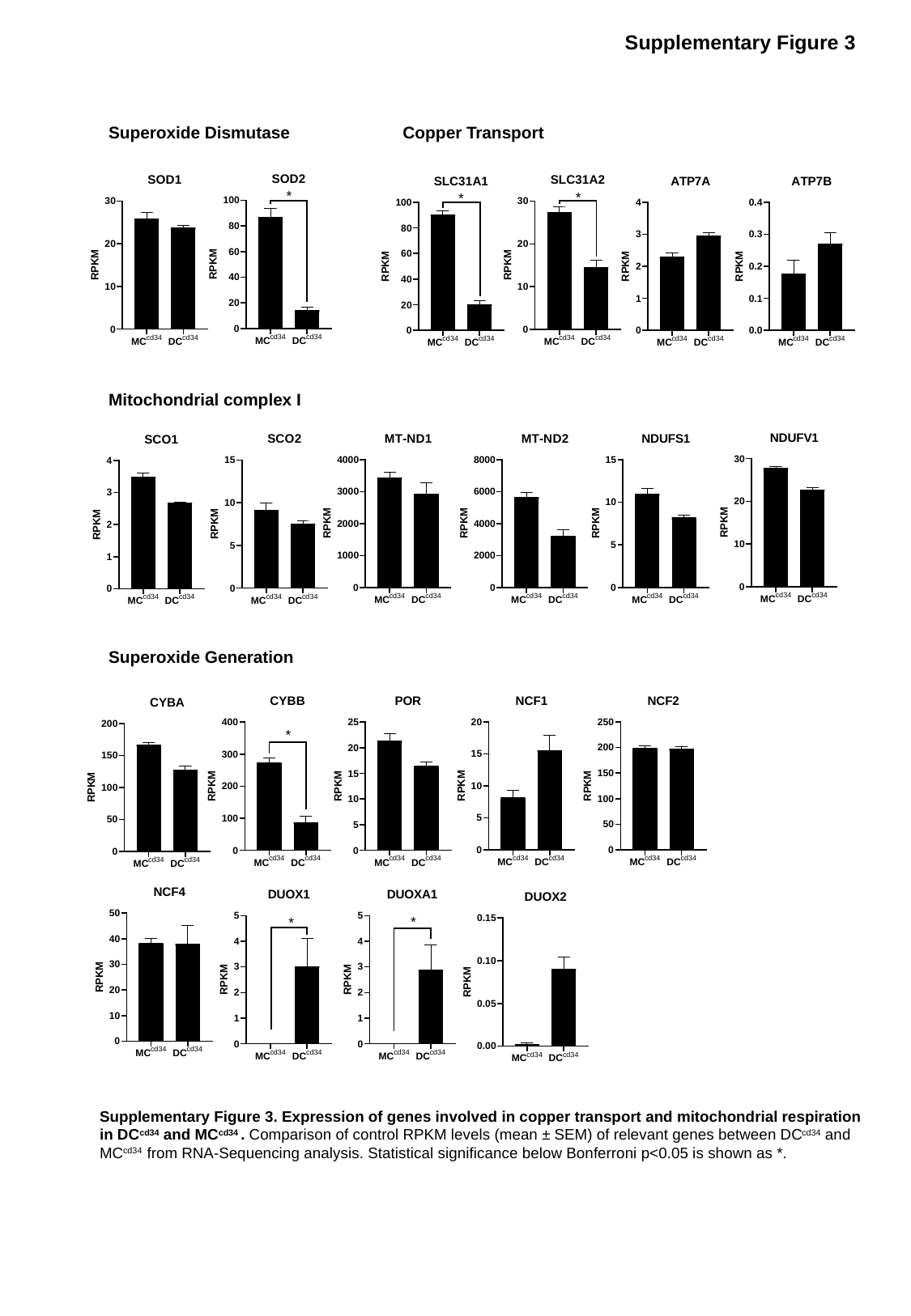

Supplementary Figure 3
Superoxide Dismutase
Copper Transport
Mitochondrial complex I
Superoxide Generation
Supplementary Figure 3. Expression of genes involved in copper transport and mitochondrial respiration in DCcd34 and MCcd34 . Comparison of control RPKM levels (mean ± SEM) of relevant genes between DCcd34 and MCcd34 from RNA-Sequencing analysis. Statistical significance below Bonferroni p<0.05 is shown as *.

## Slide 4
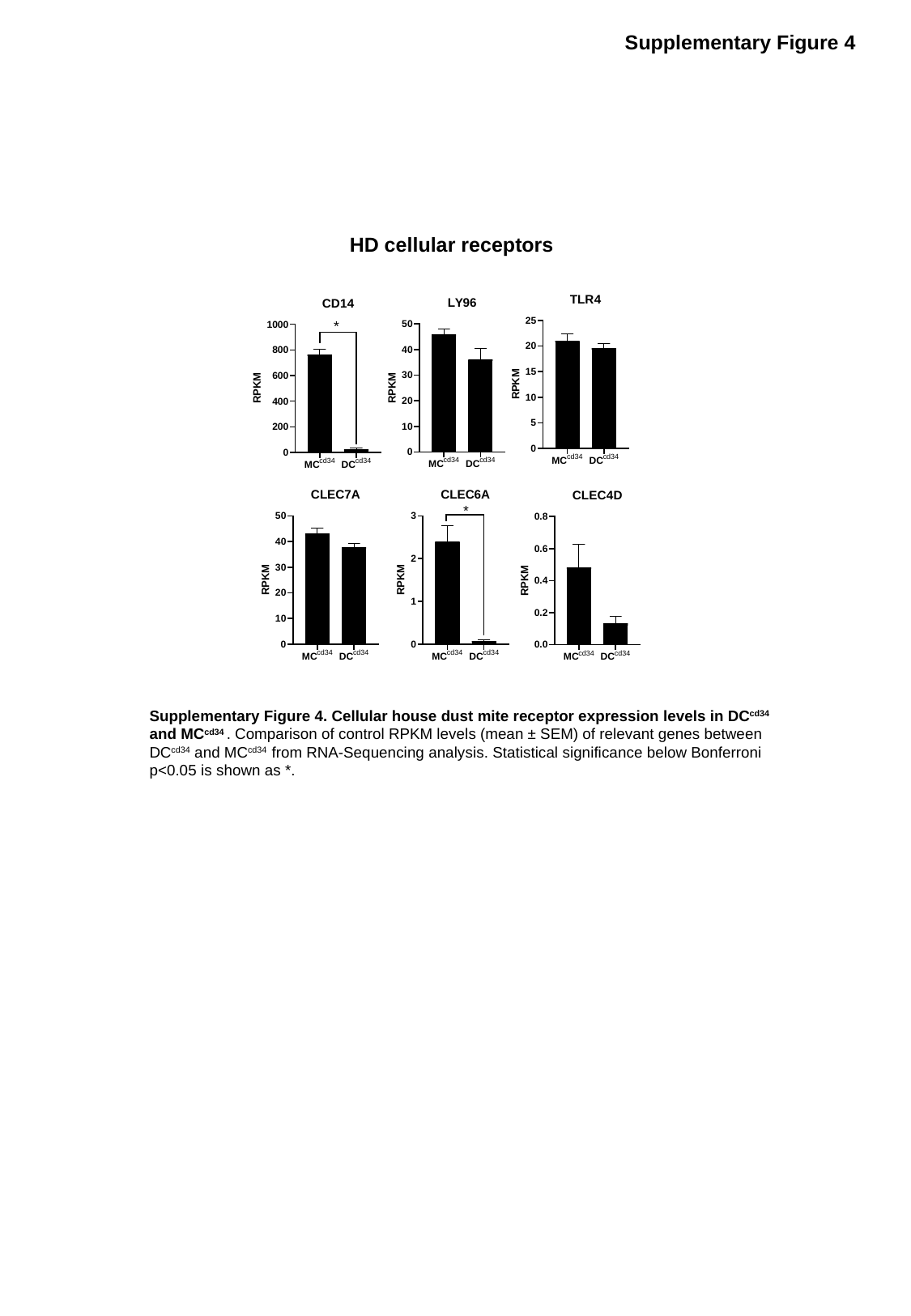

Supplementary Figure 4
HD cellular receptors
Supplementary Figure 4. Cellular house dust mite receptor expression levels in DCcd34 and MCcd34 . Comparison of control RPKM levels (mean ± SEM) of relevant genes between DCcd34 and MCcd34 from RNA-Sequencing analysis. Statistical significance below Bonferroni p<0.05 is shown as *.

## Slide 5
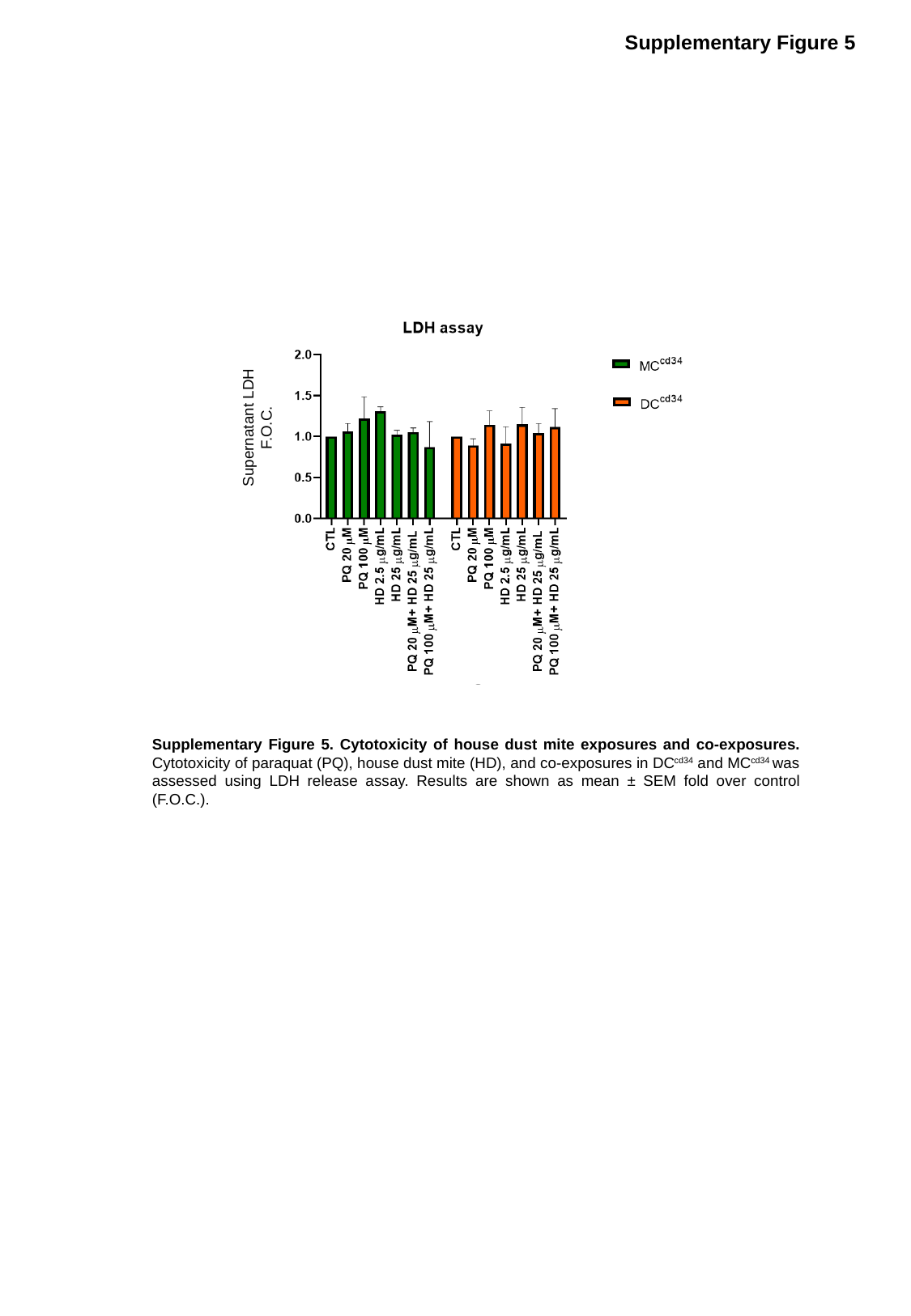

Supplementary Figure 5
Supernatant LDH F.O.C.
Supplementary Figure 5. Cytotoxicity of house dust mite exposures and co-exposures. Cytotoxicity of paraquat (PQ), house dust mite (HD), and co-exposures in DCcd34 and MCcd34 was assessed using LDH release assay. Results are shown as mean ± SEM fold over control (F.O.C.).

## Slide 6
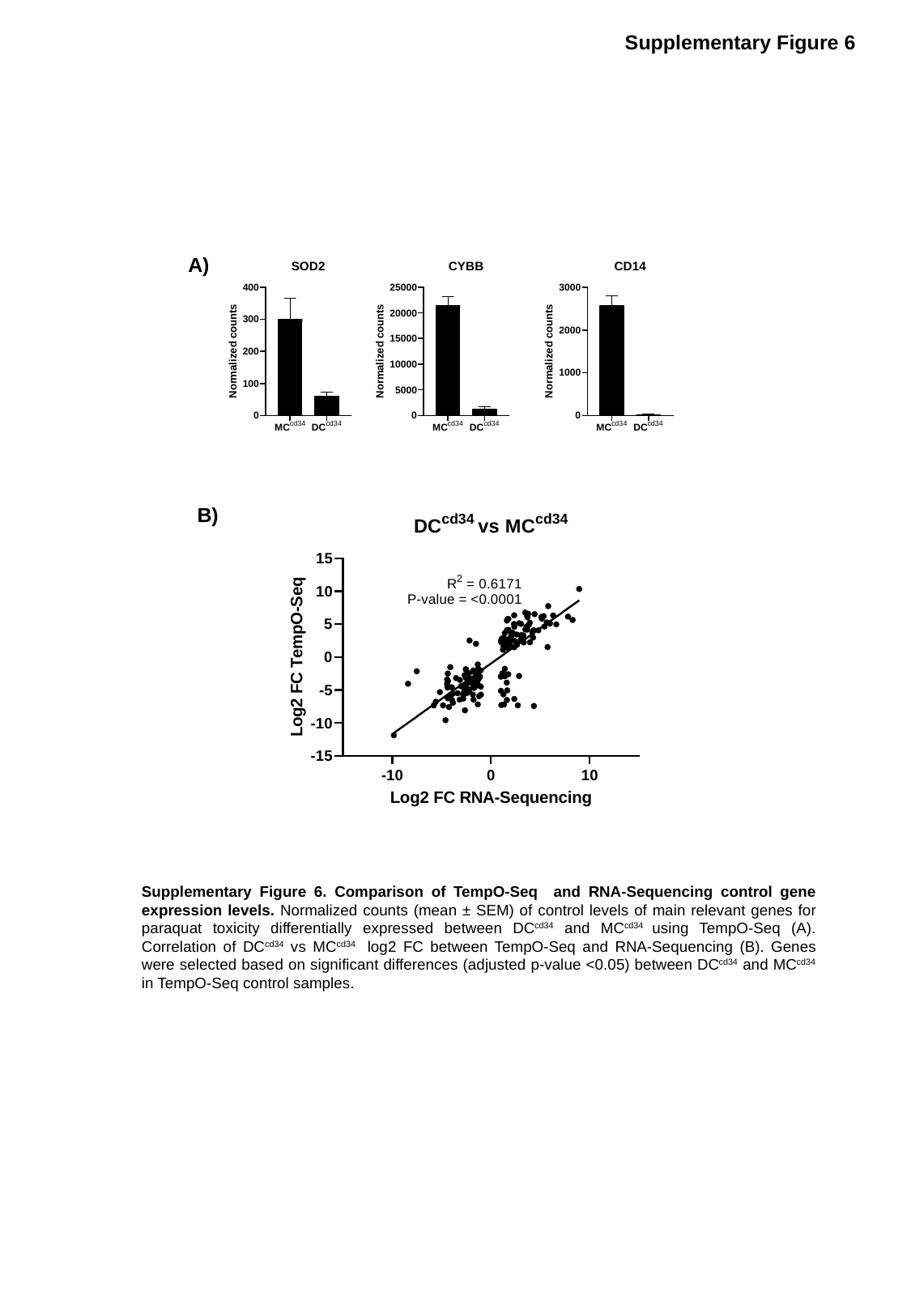

Supplementary Figure 6
A)
B)
Supplementary Figure 6. Comparison of TempO-Seq and RNA-Sequencing control gene expression levels. Normalized counts (mean ± SEM) of control levels of main relevant genes for paraquat toxicity differentially expressed between DCcd34 and MCcd34 using TempO-Seq (A). Correlation of DCcd34 vs MCcd34 log2 FC between TempO-Seq and RNA-Sequencing (B). Genes were selected based on significant differences (adjusted p-value <0.05) between DCcd34 and MCcd34 in TempO-Seq control samples.
