# Supplementary material for: CD34+ derived macrophage and dendritic cells display differential responses to paraquat
Source: Toxicol In Vitro. 2021 Sep;75:105198. doi: 10.1016/j.tiv.2021.105198 (PMC8444090; doi:10.1016/j.tiv.2021.105198)
Supplement: Supplementary file 2 — Supplementary table 1 [file mmc2.docx]

**Supplementary Table 1**

**Supplementary Table 1.** Donor information

| ID | Age | Sex | Ethnicity | Weight (kg) | Height (cm) | Smoker | Donor virus testing  (HIV-1 and 2, Hep B and C) |
| --- | --- | --- | --- | --- | --- | --- | --- |
| 1 | 27 | Male | African American | 111 | 177 | No | Negative |
| 2 | 48 | Male | Caucasian | 84 | 176 | No | Negative |
| 3 | 25 | Female | Native American | 51 | 167 | No | Negative |
| 4 | 27 | Female | Hispanic | 70 | 159 | No | Negative |
| 5 | 25 | Female | Caucasian | 72 | 179 | No | Negative |
| 6 | 36 | Male | Caucasian | 104 | 189 | No | Negative |
| 7 | 34 | Female | Caucasian | 80 | 177 | No | Negative |
| 8 | 39 | Male | Unknown | 90 | 169 | No | Negative |
| 9 | 22 | Female | Caucasian | 74 | 179 | No | Negative |
